# Body mass index, gestational weight gain and fatty acid concentrations during pregnancy: the Generation R Study

**DOI:** 10.1007/s10654-015-0106-6

**Published:** 2015-12-14

**Authors:** Aleksandra Jelena Vidakovic, Vincent W. V. Jaddoe, Olta Gishti, Janine F. Felix, Michelle A. Williams, Albert Hofman, Hans Demmelmair, Berthold Koletzko, Henning Tiemeier, Romy Gaillard

**Affiliations:** The Generation R Study Group (Na29-15), Erasmus MC, University Medical Center, PO Box 2040, 3000 CA Rotterdam, The Netherlands; Department of Pediatrics, Erasmus MC, University Medical Center, Rotterdam, The Netherlands; Department of Epidemiology, Erasmus MC, University Medical Center, Rotterdam, The Netherlands; Department of Child and Adolescent Psychiatry, Erasmus MC, University Medical Center, Rotterdam, The Netherlands; Harvard T.H. Chan School of Public Health, Boston, MA USA; Division of Metabolic Medicine, Department of Pediatrics, Dr. von Hauner Children’s Hospital, Ludwig-Maximilians-University of Munich Medical Center, Munich, Germany

**Keywords:** Body mass index, Gestational weight gain, Fatty acids, Pregnancy, Cohort

## Abstract

Obesity during pregnancy may be correlated with an adverse nutritional status affecting pregnancy and offspring outcomes. We examined the associations of prepregnancy body mass index and gestational weight gain with plasma fatty acid concentrations in mid-pregnancy. This study was embedded in a population-based prospective cohort study among 5636 women. We obtained prepregnancy body mass index and maximum weight gain during pregnancy by questionnaires. We measured concentrations of saturated fatty acid (SFA), monounsaturated fatty acid (MUFA), n-3 polyunsaturated fatty acid (n-3 PUFA) and n-6 polyunsaturated fatty acid (n-6 PUFA) at a median gestational age of 20.5 (95 % range 17.1–24.9) weeks. We used multivariate linear regression models. As compared to normal weight women, obese women had higher total SFA concentrations [difference: 0.10 standard deviation (SD) (95 % Confidence Interval (CI) 0, 0.19)] and lower total n-3 PUFA concentrations [difference: − 0.11 SD (95 % CI − 0.20, − 0.02)]. As compared to women with sufficient gestational weight gain, those with excessive gestational weight gain had higher SFA concentrations [difference: 0.16 SD (95 % CI 0.08, 0.25)], MUFA concentrations [difference: 0.16 SD (95 % CI 0.08, 0.24)] and n-6 PUFA concentrations [difference: 0.12 SD (95 % CI 0.04, 0.21)]. These results were not materially affected by adjustment for maternal characteristics. Our results suggest that obesity and excessive weight gain during pregnancy are associated with an adverse fatty acids profile. Further studies are needed to assess causality and direction of the observed associations.

**Electronic supplementary material:**

The online version of this article (doi:10.1007/s10654-015-0106-6) contains supplementary material, which is available to authorized users.

## Introduction

Overweight and obesity are major public health problems across all ages and populations in Western countries [1–8]. Previous studies reported a prevalence of up to 30 % among pregnant women [9, 10]. Obesity during pregnancy is associated with increased risks of adverse pregnancy outcomes, including gestational hypertensive disorders, gestational diabetes, fetal death and large size for gestational age infants [11–14]. Recent studies suggest that maternal obesity and excessive weight gain during pregnancy are also associated with adverse cardiovascular outcomes in the offspring [14, 15]. The mechanisms underlying these associations may involve fetal cardiovascular and metabolic adaptations in response to increased transport of specific fatty acids to the placenta and fetus [16]. Increased body mass index is associated with dyslipidemia and insulin resistance during pregnancy, which may lead to increased circulating free fatty acids concentrations [17]. Among adolescents and adults, it has been shown that a higher body mass index is associated with higher concentrations of saturated fatty acids (SFAs), lower concentrations of n-3 polyunsaturated fatty acids (PUFAs) and higher n-6/n-3 PUFA ratio [18, 19]. A previous study in the United States among 129 pregnant women suggested that women who were obese before pregnancy had lower concentrations of docosahexaenoic acid, an n-3 PUFA, and arachidonic acid, an n-6 PUFA, whereas concentrations of eicosapentaenoic acid, an n-3 PUFA, did not differ by prepregnancy body mass index categories [20]. This study did not have information on other fatty acid concentrations available.

Therefore, we examined in a population-based prospective cohort study among 5636 women, the associations of prepregnancy body mass index and gestational weight gain with plasma fatty acid concentrations in mid-pregnancy.

## Methods

### Study design

**Fig. 1:**
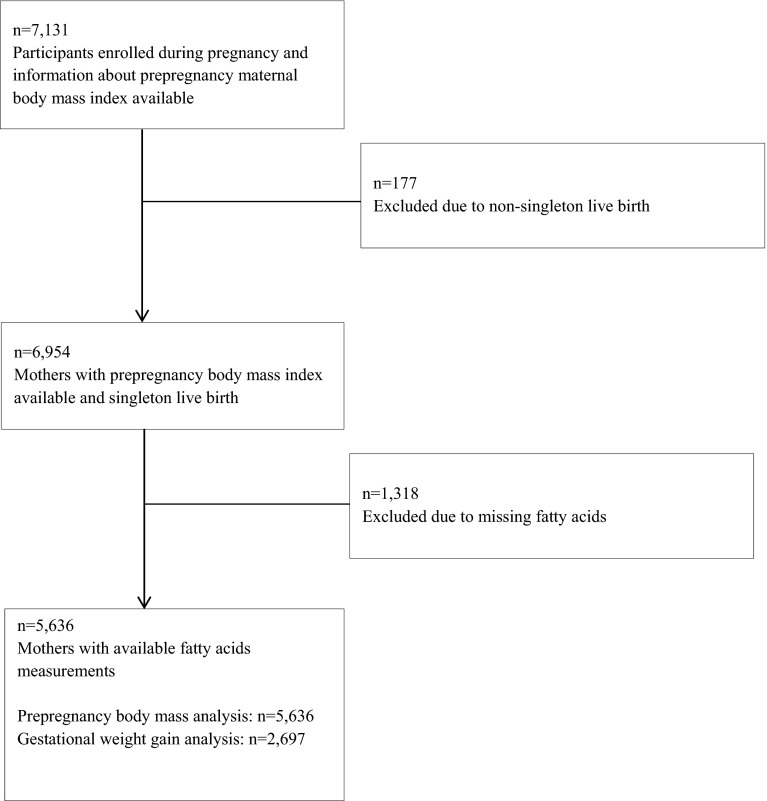
Flow chart of the participants

This study was embedded in the Generation R Study, a population-based prospective cohort study from foetal life to adulthood in Rotterdam, the Netherlands [21, 22]. The study has been approved by the Medical Ethical Committee of the Erasmus MC in Rotterdam (MEC 198.782/2001/31). All mothers gave written consent. Pregnant women with an expected delivery date from April 2002 to January 2006 were enrolled in the study. In total, 7131 mothers were enrolled during pregnancy, of whom 6954 had information on prepregnancy body mass index measurements available and gave birth to singleton live born children. Mid-pregnancy fatty acids concentrations were measured among 5636 women (Flow chart is given Fig. 1).

### Prepregnancy body mass index and gestational weight gain

At enrollment, we measured height (cm) and weight (kg) without shoes and heavy clothing, and calculated body mass index (kg/m^2^). Information about weight just before pregnancy was obtained by questionnaire. In our population for analysis, 52.3 % of all women were enrolled before a gestational age of 14 weeks. Correlation of prepregnancy weight, obtained by questionnaire, and weight measured at enrollment was 0.95 (*P* value <0.001). Prepregnancy body mass index was categorized into 4 categories (underweight [<20.0 kg/m^2^], normal weight [20.0–24.9 kg/m^2^], overweight [25.0–29.9 kg/m^2^], and obese [≥30.0 kg/m^2^]). Information about maximum weight during pregnancy was available in a subset of 2697 women and was assessed by questionnaire 2 months after delivery. According to the Institute of Medicine guidelines, we defined insufficient and excessive gestational weight gain in relation to prepregnancy body mass index (for underweight and normal weight: total weight gain <11.5 and >16.0 kg; for overweight: total weight gain <7 and >11.5 kg; for obesity: total weight gain <5 and >9.0 kg, respectively) [23].

### Fatty acid status

Venous samples were drawn at a median gestational age of 20.5 weeks (95 % range 17.1–24.9). To analyze fatty acids concentrations, EDTA plasma samples were selected and transported to the Division of Metabolic Diseases and Nutritional Medicine, Dr. von Hauner Children’s Hospital, University of Munich Medical Center. After being thawed, the analysis of plasma glycerophospholipid fatty acids was performed by a sensitive and precise high throughput method. This method is suitable for applications in large epidemiological studies [24]. Based on findings from previous studies, we selected fatty acids for our analyses, which have been associated with the risk of cardiovascular and metabolic outcomes in adults, and pregnancy outcomes [25–30]. Selected saturated fatty acids included total SFA concentrations, myristic acid (C14:0), palmitic acid (C16:0) and stearic acid (C18:0). Monounsaturated fatty acids included total MUFA concentrations, palmitoleic acid (C16:1n7) and oleic acid (C18:1n9). Polyunsaturated fatty acid included total n-3 PUFA concentrations, α-linolenic acid (ALA, C18:3n3), eicosapentaenoic acid (EPA, C20:5n3) and docosahexaenoic acid (DHA, C22:6n3), and total n-6 PUFA concentrations, linoleic acid (LA, C18:2n6), dihomo-gamma linolenic acid (DGLA, C20:3n6) and arachidonic acid (AA, C20:4n6). The ratio of total n-6/n-3 PUFA was calculated [31].

### Covariates

We obtained information on maternal prepregnancy body mass index, age, education level, ethnicity, parity, smoking, alcohol consumption and folic acid supplement by questionnaires [21]. First trimester maternal nutritional information was obtained by food frequency questionnaire [32].

### Statistical analysis

Differences in subject characteristics between body mass index categories were examined with 1-way ANOVA tests. We assessed the associations of prepregnancy body mass index, continuously and in categories, with fatty acid concentrations using linear regression models. Next, we assessed the associations of gestational weight gain, continuously and in categories, with fatty acid concentrations during pregnancy using linear regression models. To enable comparison of effect estimates, we constructed standard deviation scores (SDS) [(observed value − mean)/SD] for each fatty acid concentration. All models were adjusted for maternal age, educational level, ethnicity, parity, smoking, alcohol consumption, folic acid supplement use and total caloric and fat intake during pregnancy. We have adjusted the regression models for these maternal lifestyle factors, as they are known to be related with the main exposures and outcomes. In addition, we also adjusted the analyses for folic acid supplement use, which is known to increase plasma concentrations of docosahexaenoic acid and eicosapentaenoic acid [33]. We performed sensitivity analyses without adjusting for total energy and fat intake. In order to test if the associations of gestational weight gain with plasma fatty acid concentrations are influenced by maternal prepregnancy body mass index, we presented results from the regression models unadjusted and adjusted for maternal prepregnancy body mass index. Also, we tested potential interactions between maternal prepregnancy body mass index categories and gestational weight gain for the associations with plasma fatty acid concentrations. Since we did not observe significant interactions, we did not perform the analyses focused on gestational weight gain effects in strata of maternal prepregnancy body mass index categories. Because of the correlation between the different fatty acids, we did not apply correction for multiple testing. In order to reduce potential bias associated with missing data and to maintain statistical power, we performed multiple imputations of missing covariates by generating 5 independent datasets using the Markov Chain Monte Carlo method after which the pooled effect estimates were calculated. All analyses were performed using Statistical Package of Social Sciences version 21.0 for Windows (SPSS Inc, Chicago, IL).

## Results

### Subject characteristics

Table 1 shows that obese pregnant women tended to be younger and lower educated, as compared to normal weight women. Obese women also tended to have a lower total energy intake and were more likely to have excessive weight gain during pregnancy, compared to normal weight women. Correlations between all fatty acid concentrations are shown in Supplementary Table S1. Observed mid-pregnancy fatty acid concentrations according to body mass index are shown in Supplementary Tables S2. Table S3 shows characteristics of women according to gestational weight gain categories.

**Table 1:** Characteristics of mothers (N = 5636)

	Body mass categories					
	Total group (5636)	Underweight [<20.0 kg/m^2^] (n = 881)	Normal weight [20.0–24.9 kg/m^2^] (n = 3162)	Overweight [25.0–29.9 kg/m^2^] (n = 1094)	Obesity [≥30.0 kg/m^2^] (n = 499)	*P* value*
*Maternal characteristics*						
Age (years)	29.8 (5.2)	29.2 (5.3)	30.1 (5.2)	29.8 (5.2)	29.2 (5.0)	<0.01
Height (cm)	167.6 (7.3)	168.7 (7.0)	167.9 (7.3)	166.3 (7.5)	165.7 (7.4)	<0.01
Weight (kg)	69.2 (13.1)	56.9 (6.7)	65.7 (7.3)	77.5 (8.6)	95.2 (13.8)	<0.01
Body mass index (kg/m^2^)	23.7 (4.4)	18.9 (0.9)	22.2 (1.4)	26.9 (1.4)	33.9 (3.7)	<0.01
Education, N higher education (%)	2340 (42.9)	389 (45.3)	1508 (48.9)	360 (34.5)	83 (17.8)	<0.01
Race/ethnicity, European N (%)	3259 (58.4)	544 (62.2)	1976 (63.0)	529 (49.1)	210 (42.9)	<0.01
Parity, N nulliparous (%)	3230 (57.3)	563 (63.9)	1917 (60.7)	534 (48.8)	216 (43.3)	<0.01
Total energy intake (kcal)	2013 (587)	2061 (606)	2040 (574)	1953 (588)	1878 (602)	<0.01
Carbohydrates, energy (%)	49.1 (6.6)	49.3 (6.9)	49.3 (6.5)	48.8 (6.6)	48.1 (7.2)	0.06
Proteins, energy (%)	14.9 (2.7)	14.6 (2.8)	14.9 (2.6)	15.0 (2.7)	15.2 (3.1)	0.03
Fat, energy (%)	35.8 (5.7)	35.9 (5.9)	35.6 (5.5)	36.0 (5.6)	36.6 (6.5)	0.07
Folic acid supplement use (yes), N (%)	3354 (72.0)	535 (74.5)	1990 (66.9)	596 (66.0)	233 (68.6)	<0.01
Smoking during pregnancy (yes), N (%)	1442 (26.7)	249 (29.4)	778 (25.6)	279 (26.9)	136 (28.5)	0.03
Alcohol consumption during pregnancy (yes), N (%)	2785 (51.8)	478 (56.4)	1713 (56.5)	427 (41.6)	167 (35.5)	<0.01
Gestational weight gain (kg)	14.9 (5.7)	14.9 (5.0)	15.4 (5.1)	14.3 (6.4)	11.7 (8.1)	<0.01
*Gestational weight gain categories*						
Insufficient, N (%)	546 (20.2)	93 (21.2)	355 (21.6)	62 (13.6)	36 (22.8)	<0.01
Sufficient, N (%)	922 (34.2)	198 (45.1)	618 (37.6)	76 (16.7)	30 (19.0)	
Excessive, N (%)	1229 (45.6)	148 (33.7)	671 (40.8)	318 (69.7)	92 (58.2)	

Values represent mean (SD), median (95 % range), or number of subjects (valid %)

* Differences in subject characteristics between groups were evaluated using 1-way ANOVA test

### Saturated and monounsaturated fatty acid concentrations

Table 2 shows that a 1-SD higher body mass index was associated with 0.07 SD [95 % confidence interval (CI) 0.04, 0.10] higher total SFA concentrations, and specifically with higher palmitic acid and stearic acid concentrations, and with lower myristic acid concentrations (all *P* values <0.05). As compared to normal weight women, overweight and obese women had higher total SFA concentrations (all *P* values <0.05). A 1-SD higher gestational weight gain was associated with 0.09 SD (95 % CI 0.05, 0.13) higher total SFA concentrations, and specifically with higher myristic, palmitic and stearic acid concentrations. Total saturated fatty acid concentrations were not different between women with insufficient weight gain and with sufficient weight gain.

**Table 2:** Maternal weight during pregnancy with saturated fatty acid concentrations (N = 5636)

	Difference in saturated fatty acid (SFA) concentrations (95 % CI)			
	Total SFAs (SD)	Myristic acid (SD)	Palmitic acid (SD)	Stearic acid (SD)
*Body mass index* ^a^				
Underweight [<20.0 kg/m^2^]	−0.18 (−0.25, − 0.10)*	−0.03 (−0.10, 0.04)	−0.17 (−0.25, − 0.10)*	−0.16 (−0.24, −0.09)*
Normal weight [20.0–24.9 kg/m^2^]	Reference	Reference	Reference	Reference
Overweight [25.0–29.9 kg/m^2^]	0.08 (0.01, 0.14)*	−0.13 (−0.19, −0.06)*	0.08 (0.01, 0.15)*	0.08 (0.02, 0.15)*
Obesity [≥30.0 kg/m^2^]	0.10 (0.00, 0.19)*	−0.27 (−0.37, −0.18)*	0.13 (0.03, 0.22)*	0.07 (−0.03, 0.16)
Body mass index (SD)	0.07 (0.04, 0.10)*	−0.08 (−0.11, −0.06)*	0.08 (0.05, 0.11)*	0.06 (0.04. 0.09)*
*Gestational weight gain* ^b^				
Insufficient gestational weight gain	−0.06 (−0.16, 0.05)	−0.27 (−0.37, −0.17)*	−0.04 (−0.14, 0.06)	−0.06 (−0.16, 0.05)
Sufficient gestational weight gain	Reference	Reference	Reference	Reference
Excessive gestational weight gain	0.16 (0.08, 0.25)*	0.15 (0.07, 0.23)*	0.15 (0.06, 0.23)*	0.17 (0.09, 0.26)*
Gestational weight gain (SD)^c^	0.09 (0.05, 0.13)*	0.18 (0.14, 0.22)*	0.08 (0.04, 0.11)*	0.10 (0.06, 0.14)*
Gestational weight gain (SD)^d^	0.10 (0.06, 0.14)*	0.17 (0.13, 0.21)*	0.09 (0.05, 0.12)*	0.11 (0.07, 0.15)*

* *P* value <0.05

^a^Values are regression coefficients (95 % CI) that reflect the difference in SD of saturated fatty acid concentrations for underweight, overweight and obese women as compared to normal weight women, and per SD increase in prepregnancy body mass index. Models are adjusted for age, educational level, ethnicity, parity, smoking and alcohol consumption, folic acid supplement use and total caloric and fat intake during pregnancy

^b^Values are regression coefficients (95 % CI) that reflect the difference in SD of saturated fatty acid concentrations for insufficient weight gain and excessive weight gain women as compared to women with sufficient weight gain, and per SD increase in weight gain. Models are adjusted for age, educational level, ethnicity, parity, smoking and alcohol consumption, folic acid supplement use and total caloric and fat intake during pregnancy. Models for weight gain defined according to the IOM criteria are additionally adjusted for prepregnancy body mass index

^c^Gestational weight gain SD unadjusted for body mass index

^d^Gestational weight gain SD adjusted for body mass index

Table 3 shows that body mass index was not associated with total MUFA and oleic acid concentrations. However, a 1-SD higher body mass index was associated with 0.08 SD (95 % CI 0.06, 0.11) higher palmitoleic acid concentrations. A 1-SD higher gestational weight gain was associated with 0.10 SD (95 % CI 0.06, 0.13) higher total MUFA concentrations and all specific MUFA concentrations. Compared to women with sufficient weight gain, those with excessive weight gain had higher total MUFA, palmitoleic and oleic acid concentrations (all *P* values <0.05).

**Table 3:** Maternal weight during pregnancy with monounsaturated fatty acid concentrations (N = 5636)

	Difference in monounsaturated fatty acids (MUFAs) concentrations (95 % CI)		
	Total MUFAs (SD)	Palmitoleic acid (SD)	Oleic acid (SD)
*Body mass index* ^a^			
Underweight [<20.0 kg/m^2^]	−0.05 (−0.12, 0.02)	−0.12 (−0.19, −0.05)*	−0.03 (−0.10, 0.04)
Normal weight [20.0–24.9 kg/m^2^]	Reference	Reference	Reference
Overweight [25.0–29.9 kg/m^2^]	−0.02 (−0.08, 0.05)	0.06 (−0.01, 0.13)	−0.03 (−0.10, 0.04)
Obesity [≥30.0 kg/m^2^]	−0.02 (−0.11, 0.07)	0.22 (0.13, 0.32)*	−0.07 (−0.16, 0.02)
Body mass index (SD)	0 (−0.02, 0.03)	0.08 (0.06, 0.11)*	−0.01 (−0.04, 0.01)
Gestational weight gain^b^			
Insufficient gestational weight gain	−0.04 (−0.14, 0.06)	−0.12 (−0.22, −0.01)*	−0.04 (−0.14, 0.06)
Sufficient gestational weight gain	Reference	Reference	Reference
Excessive gestational weight gain	0.16 (0.08, 0.24)*	0.18 (0.10, 0.27)*	0.17 (0.08, 0.24)*
Gestational weight gain (SD)^c^	0.10 (0.06, 0.13)*	0.13 (0.10, 0.17)*	0.10 (0.06, 0.14)*
Gestational weight gain (SD)^d^	0.10 (0.06, 0.14)*	0.15 (0.11, 0.19)*	0.10 (0.06, 0.14)*

* *P* value <0.05

^a^Values are regression coefficients (95 % CI) that reflect the difference in SD of monounsaturated fatty acid concentrations for underweight, overweight and obese women as compared to normal weight women, and per SD increase in prepregnancy body mass index. Models are adjusted for age, educational level, ethnicity, parity, smoking and alcohol consumption, folic acid supplement use and total caloric and fat intake during pregnancy

^b^Values are regression coefficients (95 % CI) that reflect the difference in SD of monounsaturated fatty acid concentrations for insufficient weight gain and excessive weight gain women as compared to women with sufficient weight gain, and per SD increase in weight gain. Models are adjusted for age, educational level, ethnicity, parity, smoking and alcohol consumption, folic acid supplement use and total caloric and fat intake during pregnancy. Models for weight gain defined according to the IOM criteria are additionally adjusted for prepregnancy body mass index

^c^Gestational weight gain SD unadjusted for body mass index

^d^Gestational weight gain SD adjusted for body mass index

### N-3 and n-6 polyunsaturated fatty acid concentrations

Table 4 shows that higher body mass index was not associated with total n-3 PUFA and docosahexaenoic acid concentrations, but was associated with lower α-linolenic acid and eicosapentaenoic acid concentrations (all *P* values <0.05). As compared to normal weight women, underweight women tended to have lower total n-3 PUFA and all specific n-3 PUFA concentrations (all *P* values <0.05). Higher gestational weight gain was not associated with n-3 PUFA and docosahexaenoic acid concentrations, but was associated with higher α-linolenic acid eicosapentaenoic acid concentrations. None of the n-3 PUFA concentrations were different between women with excessive weight gain as compared to those with sufficient weight gain.

**Table 4:** Maternal weight during pregnancy with n-3 polyunsaturated fatty acid concentrations (N = 5636)

	Difference in n-3 Polyunsaturated fatty acids (PUFAs) concentrations (95 % CI)			
	Total n-3 PUFAs (SD)	α-Linolenic acid (SD)	Eicosapentaenoic acid (SD)	Docosahexaenoic acid (SD)
*Body mass index* ^a^				
Underweight [<20.0 kg/m^2^]	−0.11 (−0.18, −0.05)*	0.06 (−0.01, 0.13)	−0.03 (−0.10, 0.04)	−0.13 (−0.20, −0.06)*
Normal weight [20.0–24.9 kg/m^2^]	Reference	Reference	Reference	Reference
Overweight [25.0–29.9 kg/m^2^]	−0.04 (−0.11, 0.02)	−0.17 (−0.24, −0.11)*	−0.05 (−0.11, 0.02)	−0.01 (−0.07, 0.06)
Obesity [≥30.0 kg/m^2^]	−0.11 (−0.20, −0.02)*	−0.32 (−0.41, −0.23)*	−0.10 (−0.19, −0.01)*	−0.05 (−0.14, 0.04)
Body mass index (SD)	−0.02 (−0.04, 0.01)	−0.12 (−0.15, −0.09)*	−0.03 (−0.05, −0.01)*	0.01 (−0.02, 0.03)
*Weight gain* ^b^				
Insufficient gestational weight gain	−0.07 (−0.17, 0.03)	−0.14 (−0.24, −0.04)*	−0.18 (−0.28, −0.07)*	−0.01 (−0.11, 0.09)
Sufficient gestational weight gain	Reference	Reference	Reference	Reference
Excessive gestational weight gain	0.01 (−0.08, 0.09)	0.05 (−0.03, 0.14)	0.02 (−0.07, 0.11)	−0.02 (−0.11, 0.06)
Gestational weight gain (SD)^c^	0.03 (−0.01, 0.06)	0.09 (0.05, 0.13)*	0.08 (0.04, 0.12)*	−0.02 (−0.06, 0.02)
Gestational weight gain (SD)^d^	0.02 (−0.02, 0.06)	0.07 (0.04, 0.11)*	0.08 (0.04, 0.12)*	−0.02 (−0.06, 0.02)

* *P* value <0.05

^a^Values are regression coefficients (95 % CI) that reflect the difference in SD of n-3 PUFA concentrations for underweight, overweight and obese women as compared to normal weight women, and per SD increase in prepregnancy body mass index. Models are adjusted for, age, educational level, ethnicity, parity, smoking and alcohol consumption, folic acid supplement use and total caloric and fat intake during pregnancy

^b^Values are regression coefficients (95 % CI) that reflect the difference in SD of n-3 PUFA concentrations for insufficient weight gain and excessive weight gain women as compared to women with sufficient weight gain, and per SD increase in weight gain. Models are adjusted for, age, educational level, ethnicity, parity, smoking and alcohol consumption, folic acid supplement use and total caloric and fat intake during pregnancy. Models for weight gain defined according to the IOM criteria are additionally adjusted for prepregnancy body mass index

^c^Gestational weight gain SD unadjusted for body mass index

^d^Gestational weight gain SD adjusted for body mass index

Table 5 shows that 1-SD higher body mass index was associated with 0.06 SD (95 % CI 0, 0.09) higher total n-6 PUFAs, and specifically with higher dihomo-gamma linoelinic acid and arachidonic acid, but with lower linoleic acid concentrations (all *P* values <0.05). Compared to normal weight women, obese women had lower linoleic acid concentrations, but higher dihomo-gamma and arachidonic acid concentrations (all *P* values <0.05). A 1-SD higher gestational weight gain was associated with 0.04 SD (95 % CI 0.01, 0.08) higher total n-6 PUFAs and specifically with higher dihomo-gamma linoelinic acid concentrations. As compared to women with sufficient weight gain, those with excessive weight gain had higher n-6 PUFA concentrations (*P* values <0.05). Only linoleic acid concentrations were not different between normal weight and obese women.

**Table 5:** Maternal weight during pregnancy with n-6 polyunsaturated fatty acid concentrations (N = 5636)

	Difference in n-6 Polyunsaturated fatty acids (PUFAs) concentrations (95 % CI)			
	Total n-6 PUFAs (SD)	Linoleic acid (SD)	Dihomo-gamma linolenic acid (SD)	Arachidonic acid (SD)
*Body mass index* ^a^				
Underweight [<20.0 kg/m^2^]	−0.17 (−0.24, −0.09)*	−0.03 (−0.11, 0.04)	−0.27 (−0.34, −0.20)*	−0.23 (−0.30, −0.16)*
Normal weight [20.0–24.9 kg/m^2^]	Reference	Reference	Reference	Reference
Overweight [25.0–29.9 kg/m^2^]	0.09 (0.02, 0.16)*	−0.03 (−0.09, 0.04)	0.16 (0.10, 0.23)*	0.23 (0.16, 0.30)*
Obesity [≥30.0 kg/m^2^]	0.06 (−0.04, 0.15)	−0.23 (−0.32, −0.13)*	0.35 (0.26, 0.45)*	0.45 (0.35, 0.54)*
Body mass index (SD)	0.06 (0, 0.09)*	−0.05 (−0.08, −0.02)*	0.16 (0.14, 0.19)*	0.19 (0.17, 0.22)*
*Weight gain* ^b^				
Insufficient gestational weight gain	0.02 (−0.08, 0.12)	0.02 (−0.09, 0.12)	−0.08 (−0.18, 0.02)	0.07 (−0.04, 0.17)
Sufficient gestational weight gain	Reference	Reference	Reference	Reference
Excessive gestational weight gain	0.12 (0.04, 0.21)*	0.05 (−0.04, 0.13)	0.29 (0.21, 0.37)*	0.05 (−0.03, 0.13)
Gestational weight gain (SD)^c^	0.04 (0.01, 0.08)*	0.02 (−0.02, 0.06)	0.15 (0.11, 0.19)*	−0.03 (−0.06, 0.01)
Gestational weight gain (SD)^d^	0.05 (0.02, 0.09)*	0.01 (−0.02, 0.05)	0.18 (0.14, 0.22)*	−0.00 (−0.04, 0.04)

* *P* value <0.05

^a^Values are regression coefficients (95 % CI) that reflect the difference in SD of n-6 PUFA concentrations for underweight, overweight and obese women as compared to normal weight women, and per SD increase in prepregnancy body mass index. Models are adjusted for age, educational level, ethnicity, parity, smoking and alcohol consumption, folic acid supplement use and total caloric and fat intake during pregnancy

^b^Values are regression coefficients (95 % CI) that reflect the difference in SD of n-6 PUFA concentrations for insufficient weight gain and excessive weight gain women as compared to women with sufficient weight gain, and per SD increase in weight gain. Models are adjusted for age, educational level, ethnicity, parity, smoking and alcohol consumption, folic acid supplement use and total caloric and fat intake during pregnancy. Models for weight gain defined according to the IOM criteria are additionally adjusted for prepregnancy body mass index

^c^Gestational weight gain SD unadjusted for body mass index

^d^Gestational weight gain SD adjusted for body mass index

**Fig. 2:**
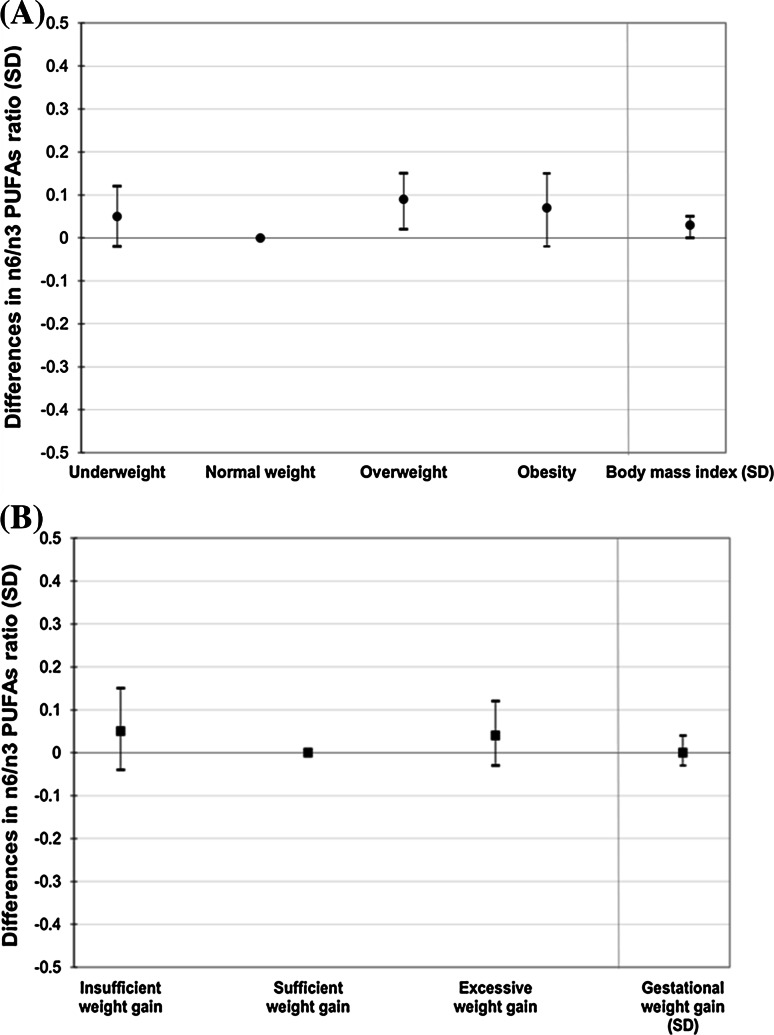
Maternal body mass index (**a**) and gestational weight gain (**b**) with n-6/n-3 PUFA ratio (N = 5636). **a** Values are regression coefficients (95 % CI) that reflect the difference in SD of n-6/n-3 PUFAs ratio for underweight, overweight and obese women as compared to normal weight women, and per SD increase in prepregnancy body mass index. *PUFAs* Polyunsaturated fatty acids. Models are adjusted for maternal age, educational level, ethnicity, parity, smoking and alcohol consumption, folic acid supplement use and total caloric and fat intake during pregnancy. *P value <0.05. **b** Values are regression coefficients (95 % CI) that reflect the difference in SD of n-6/n-3 PUFAs ratio for insufficient weight gain, and excessive weight gain women as compared to women with sufficient weight gain, and per SD increase in weight gain. Models are adjusted for age, educational level, ethnicity, parity, smoking and alcohol consumption, folic acid supplement use and total caloric and fat intake during pregnancy. Models for weight gain are additionally adjusted for prepregnancy body mass index. *P value <0.05

Figure 2 shows that as compared to normal weight women, those with obesity tended to have a 0.07 SD (95 % CI − 0.02, 0.15) higher n-6/n-3 PUFA ratio. Also, as compared to women with sufficient weight gain, those with excessive weight gain had a 0.06 SD (95 % CI − 0.01, 0.14) higher n-6/n-3 PUFA ratio.

### Additional analysis

Similar results were observed when we did not adjust the gestational weight gain analyses for maternal prepregnancy body mass index (Results given in Supplementary Tables S4 and S5). We observed similar results for all analyses without adjustment for total energy and fat intake (Supplementary Tables S6–S9). The results from the unadjusted models are given in Supplementary Tables S10–S13. Results were not materially different between the adjusted and unadjusted models.

## Discussion

In this population-based cohort study, we observed that higher prepregnancy body mass index was associated with higher total SFA and total n-6 PUFA concentrations. Also, we observed that higher gestational weight gain was, independent of prepregnancy body mass index, associated with higher total SFA, MUFA and n-6 PUFA concentrations.

### Methodological considerations

This study was embedded in a population-based prospective cohort with a large number of subjects. To the best of our knowledge, the current study is the largest population-based study focused on the associations of body mass index and weight gain during gestation with fatty acid concentrations. However, some limitations need to be discussed. Within this study, we correlated prepregnancy body mass index and maximum weight gain with mid-pregnancy fatty acid concentrations. The timing of these measurements restricts us for drawing conclusions about the direction of any association. However, fatty acid patterns in plasma phospholipids show a relatively high degree of tracking over time [34, 35]. Information on maternal prepregnancy weight and maximum weight was self-reported. We observed high correlations between weight in early and late pregnancy with prepregnancy weight and maximum weight, respectively, but self-reported weight might be underestimated. We measured a large number of fatty acid concentrations in plasma samples only once during pregnancy. No information was available about fatty acid concentrations in other trimesters. Fatty acids measured in plasma may reflect a time frame of dietary intake of approximately 2 weeks and seem to be reasonable indicators for the recent intake [36]. Unfortunately, no information was available about erythrocyte lipid levels, which would reflect a longer intake period. We assessed maternal dietary intake during first trimester by a food frequency questionnaire (FFQ) and adjusted the main analyses for maternal total energy and fat intake. No differences in results were observed with and without adjustment for total energy and fat intake. Unfortunately, these FFQs were not validated for assessing maternal dietary fatty acids intake. Further observational and experimental studies are needed to explore the associations of maternal dietary fatty acids intake with maternal body mass index and gestational weight gain. Although, we were able to adjust our analyses for various potential confounders, residual confounding might still be an issue as in any observational study.

### Interpretation of main findings

Overweight and obesity during pregnancy are important public health problems and are associated with adverse maternal and neonatal outcomes [9, 37]. Multiple previous studies have reported that higher prepregnancy body mass index and excessive gestational weight gain are associated with increased risks of gestational diabetes, neonatal mortality, and obesity in offspring [11, 12, 15, 37–39]. The mechanism linking higher maternal obesity and excessive gestational weight gain with offspring outcomes may include fetal overnutrition [16]. Fetal exposure to increased concentrations of free fatty acids may lead to intrauterine metabolic adaptations and disproportionate fetal growth [40]. Increased concentrations of free fatty acids during pregnancy are associated with childhood obesity [41, 42]. Previously, we have shown that higher maternal n-3 PUFA and lower n-6 PUFA concentrations in pregnancy are also associated with lower childhood systolic blood pressure [43].

Results from previous studies support associations of obesity with fatty acid concentrations. A study among 124 adults in Australia showed that obese subjects had lower plasma n-3 PUFA concentrations [19]. A systematic review based on 21 studies showed that overweight or obese adults had lower linoleic acid and higher dihomo-gamma-linolenic acid, n-6 PUFAs [44]. A study among 120 adolescents aged 12 years old from France showed that overweight adolescents had higher SFA concentrations and lower total n-3 PUFA concentrations [45]. Only one study has assessed the associations of body mass index with fatty acid concentrations among pregnant women [20]. This study among 129 pregnant women in the US showed that as compared with lean women, obese women had lower docosahexaenoic acid, an n-3 PUFA, and arachidonic, an n-6 PUFA, concentrations during pregnancy [20]. No information about other fatty acid concentrations was available. In line with these previous results, we observed that higher body mass index was associated with higher total n-6 PUFA concentrations. Obese women had lower linoleic acid concentrations, but higher dihomo-gamma and arachidonic acid concentrations. All n-3 PUFA concentrations tended to be lower among obese women with the strongest association for α-linolenic acid. For the SFAs, obese women had lower myristic acid concentrations and higher palmitic acid concentrations, whereas for the MUFAs, obese women had higher palmitoleic acid concentrations. These results suggest that the associations of body mass index with fatty acid concentrations in pregnant women are in line with those observed in non-pregnant adults.

We also assessed the associations of gestational weight gain with fatty acid concentrations. Gestational weight gain is a complex measure, which reflects maternal fat accumulation, fluid and blood volume expansion, and fetal and placental growth [23]. Excessive gestational weight gain is defined as increased weight gain between the time of conception and the onset of labor and is a related with pregnancy and offspring outcomes [14]. We observed that total SFA concentrations, specifically myristic, palmitic and stearic acids, were higher among women with excessive gestational weight gain. Excessive gestational weight gain was also associated with total MUFA concentrations, specifically palmitoleic and oleic acids. We observed that excessive gestational weight gain was associated with higher concentrations of total n-6 PUFA, specifically dihomo-gamma linoleic and arachidonic acids, but no association was observed with n-3 PUFA concentrations. The observed associations of excessive gestational weight gain with fatty acid concentrations were present with and without adjustment of body mass index. Thus, women with excessive gestational weight gain during pregnancy have, independent of their prepregnancy body mass index, an adverse fatty acid profile.

Our results suggest that pregnant women with either a high body mass index or high gestational weight gain have an adverse fatty acids profile, characterized by higher concentrations of SFA, MUFA and n-6 PUFA, and lower concentrations of n-3 PUFA. From our study, it is difficult to determine whether body mass index and gestational weight gain cause adverse fatty acid profiles, or whether the direction of the association is reversed. It has been suggested that higher n-6 PUFA concentrations lead to higher adipose tissue development through promotion of preadipocyte differentiation and that higher n-3 PUFA concentrations decreases adipose tissue mass and suppresses development of obesity [46, 47]. Also, saturated fatty acids and the monounsaturated fatty acids stimulate adipocyte differentiation and triacylglycerol accumulation [48]. In the other direction, obesity alters adipose tissue metabolic and endocrine function and leads to an increased release of fatty acids, affecting subsequent adiposity and thus creating a vicious cycle [49]. Further studies are needed to explore the causality and direction of the observed associations. Studies with longitudinal measurements of both weight and fatty acid concentrations before and during pregnancy may help to identify the direction of these associations. Also, experimental and Mendelian Randomization studies may help to assess the causality of the observed associations [50, 51].

## Conclusion

We observed that higher prepregnancy body mass index was associated with higher total SFA and total n-6 PUFA concentrations, whereas higher gestational weight gain was associated with higher total SFA, MUFA and n-6 PUFA concentrations. Further observational and experimental studies are needed for replication and to explore the direction and underlying mechanism of these associations.

## Electronic supplementary material

Supplementary material 1 (DOCX 69 kb)

## References

[1] Haslam DW, James WP (2005). Obesity. Lancet.

[2] Liu XM, Liu YJ, Zhan J, He QQ (2015). Overweight, obesity and risk of all-cause and cardiovascular mortality in patients with type 2 diabetes mellitus: a dose-response meta-analysis of prospective cohort studies. Eur J Epidemiol.

[3] O’Doherty MG, Jorgensen T, Borglykke A (2014). Repeated measures of body mass index and C-reactive protein in relation to all-cause mortality and cardiovascular disease: results from the consortium on health and ageing network of cohorts in Europe and the United States (CHANCES). Eur J Epidemiol.

[4] Niedziela J, Hudzik B, Niedziela N (2014). The obesity paradox in acute coronary syndrome: a meta-analysis. Eur J Epidemiol.

[5] Horvei LD, Braekkan SK, Mathiesen EB, Njolstad I, Wilsgaard T, Hansen JB (2014). Obesity measures and risk of venous thromboembolism and myocardial infarction. Eur J Epidemiol.

[6] Song X, Pukkala E, Dyba T (2014). Body mass index and cancer incidence: the FINRISK study. Eur J Epidemiol.

[7] Etemadi A, Abnet CC, Kamangar F (2014). Impact of body size and physical activity during adolescence and adult life on overall and cause-specific mortality in a large cohort study from Iran. Eur J Epidemiol.

[8] Hjellvik V, Selmer R, Gjessing HK, Tverdal A, Vollset SE (2013). Body mass index, smoking, and risk of death between 40 and 70 years of age in a Norwegian cohort of 32,727 women and 33,475 men. Eur J Epidemiol.

[9] Huda SS, Brodie LE, Sattar N (2010). Obesity in pregnancy: prevalence and metabolic consequences. Semin Fetal Neonatal Med.

[10] Callaway LK, Prins JB, Chang AM, McIntyre HD (2006). The prevalence and impact of overweight and obesity in an Australian obstetric population. Med J Aus..

[11] Starling AP, Brinton JT, Glueck DH (2015). Associations of maternal BMI and gestational weight gain with neonatal adiposity in the Healthy Start study. Am J Clin Nutr.

[12] Kongubol A, Phupong V (2011). Prepregnancy obesity and the risk of gestational diabetes mellitus. BMC Pregnancy Childbirth.

[13] Driul L, Cacciaguerra G, Citossi A, Martina MD, Peressini L, Marchesoni D (2008). Prepregnancy body mass index and adverse pregnancy outcomes. Arch Gynecol Obstet.

[14] Gaillard R (2015). Maternal obesity during pregnancy and cardiovascular development and disease in the offspring. Eur J Epidemiol.

[15] Gaillard R, Steegers EA, Franco OH, Hofman A, Jaddoe VW (2015). Maternal weight gain in different periods of pregnancy and childhood cardio-metabolic outcomes. The Generation R Study. Int J Obes (Lond).

[16] Lawlor DA, Smith GD, O’Callaghan M (2007). Epidemiologic evidence for the fetal overnutrition hypothesis: findings from the mater-university study of pregnancy and its outcomes. Am J Epidemiol.

[17] Nelson SM, Matthews P, Poston L (2010). Maternal metabolism and obesity: modifiable determinants of pregnancy outcome. Hum Reprod Updat.

[18] Karlsson M, Marild S, Brandberg J, Lonn L, Friberg P, Strandvik B (2006). Serum phospholipid fatty acids, adipose tissue, and metabolic markers in obese adolescents. Obesity (Silver Spring).

[19] Micallef M, Munro I, Phang M, Garg M (2009). Plasma n-3 Polyunsaturated Fatty Acids are negatively associated with obesity. Br J Nutr.

[20] Tomedi LE, Chang CC, Newby PK (2013). Pre-pregnancy obesity and maternal nutritional biomarker status during pregnancy: a factor analysis. Public Health Nutr.

[21] Jaddoe VW, van Duijn CM, Franco OH (2012). The Generation R Study: design and cohort update 2012. Eur J Epidemiol.

[22] Kruithof CJ, Kooijman MN, van Duijn CM (2014). The Generation R Study: biobank update 2015. Eur J Epidemiol.

[23] Institute of Medicine (US) and National Research Council (US) Committee to Reexamine IOM Pregnancy Weight Guidelines, Rasmussen KM, Yaktine AL, editors. Weight gain during pregnancy: reexamining the guidelines. Washington, DC: National Academies Press; 2009. 20669500

[24] Glaser C, Demmelmair H, Koletzko B (2010). High-throughput analysis of fatty acid composition of plasma glycerophospholipids. J Lipid Res.

[25] Wang L, Manson JE, Rautiainen S (2015). A prospective study of erythrocyte polyunsaturated fatty acid, weight gain, and risk of becoming overweight or obese in middle-aged and older women. Eur J Nutr.

[26] Kris-Etherton PM (1999). AHA, science advisory. Monounsaturated fatty acids and risk of cardiovascular disease. American Heart Association. Nutrition Committee. Circulation.

[27] Markey O, Vasilopoulou D, Givens DI, Lovegrove JA (2014). Dairy and cardiovascular health: friend or foe?. Nutr Bull.

[28] Chen X, Scholl TO, Leskiw M, Savaille J, Stein TP (2010). Differences in maternal circulating fatty acid composition and dietary fat intake in women with gestational diabetes mellitus or mild gestational hyperglycemia. Diabetes Care.

[29] Jensen CL (2006). Effects of n-3 fatty acids during pregnancy and lactation. Am J Clin Nutr.

[30] Qiu C, Sanchez SE, Larrabure G, David R, Bralley JA, Williams MA (2006). Erythrocyte omega-3 and omega-6 polyunsaturated fatty acids and preeclampsia risk in Peruvian women. Arch Gynecol Obstet.

[31] Steenweg-de Graaff JC, Tiemeier H, Basten MG (2015). Maternal LC-PUFA status during pregnancy and child problem behavior: the Generation R Study. Pediatr Res.

[32] Heppe DH, Medina-Gomez C, Hofman A, Franco OH, Rivadeneira F, Jaddoe VW (2013). Maternal first-trimester diet and childhood bone mass: the Generation R Study. Am J Clin Nutr.

[33] Das UN (2008). Folic acid and polyunsaturated fatty acids improve cognitive function and prevent depression, dementia, and Alzheimer’s disease–but how and why?. Prostaglandins Leukot Essent Fatty Acids.

[34] Zeleniuch-Jacquotte A, Chajes V, Van Kappel AL, Riboli E, Toniolo P (2000). Reliability of fatty acid composition in human serum phospholipids. Eur J Clin Nutr.

[35] Guerra A, Demmelmair H, Toschke AM, Koletzko B (2007). Three-year tracking of fatty acid composition of plasma phospholipids in healthy children. Ann Nutr Metab.

[36] Skeaff CM, Hodson L, McKenzie JE (2006). Dietary-induced changes in fatty acid composition of human plasma, platelet, and erythrocyte lipids follow a similar time course. J Nutr.

[37] Gaillard R, Felix JF, Duijts L, Jaddoe VW (2014). Childhood consequences of maternal obesity and excessive weight gain during pregnancy. Acta Obstet Gynecol Scand.

[38] Johansson S, Villamor E, Altman M, Bonamy AK, Granath F, Cnattingius S (2014). Maternal overweight and obesity in early pregnancy and risk of infant mortality: a population based cohort study in Sweden. BMJ.

[39] Lau EY, Liu J, Archer E, McDonald SM, Liu J (2014). Maternal weight gain in pregnancy and risk of obesity among offspring: a systematic review. J Obes.

[40] Schaefer-Graf UM, Graf K, Kulbacka I (2008). Maternal lipids as strong determinants of fetal environment and growth in pregnancies with gestational diabetes mellitus. Diabetes Care.

[41] Poston L (2012). Maternal obesity, gestational weight gain and diet as determinants of offspring long term health. Best Pract Res Clin Endocrinol Metab.

[42] Moon RJ, Harvey NC, Robinson SM (2013). Maternal plasma polyunsaturated fatty acid status in late pregnancy is associated with offspring body composition in childhood. J Clin Endocrinol Metab.

[43] Vidakovic AJ, Gishti O, Steenweg-de Graaff J (2015). Higher maternal plasma n-3 PUFA and lower n-6 PUFA concentrations in pregnancy are associated with lower childhood systolic blood pressure. J Nutr.

[44] Fekete K, Gyorei E, Lohner S, Verduci E, Agostoni C, Decsi T (2015). Long-chain polyunsaturated fatty acid status in obesity: a systematic review and meta-analysis. Obes Rev.

[45] Klein-Platat C, Drai J, Oujaa M, Schlienger JL, Simon C (2005). Plasma fatty acid composition is associated with the metabolic syndrome and low-grade inflammation in overweight adolescents. Am J Clin Nutr.

[46] Buckley JD, Howe PR (2009). Anti-obesity effects of long-chain omega-3 polyunsaturated fatty acids. Obes Rev.

[47] Muhlhausler BS, Ailhaud GP (2013). Omega-6 polyunsaturated fatty acids and the early origins of obesity. Curr Opin Endocrinol Diabetes Obes.

[48] Madsen L, Petersen RK, Kristiansen K (2005). Regulation of adipocyte differentiation and function by polyunsaturated fatty acids. Biochim Biophys Acta.

[49] Kishino T, Watanabe K, Urata T (2008). Visceral fat thickness in overweight men correlates with alterations in serum fatty acid composition. Clin Chim Acta.

[50] Smith GD, Ebrahim S (2004). Mendelian randomization: prospects, potentials, and limitations. Int J Epidemiol.

[51] Smith GD, Ebrahim S (2003). ‘Mendelian randomization’: can genetic epidemiology contribute to understanding environmental determinants of disease?. Int J Epidemiol.

