# Supplementary material for: Body mass index, gestational weight gain and fatty acid concentrations during pregnancy: the Generation R Study
Source: Eur J Epidemiol. 2015 Dec 14;30:1175–85. doi: 10.1007/s10654-015-0106-6 (PMC4684831; doi:10.1007/s10654-015-0106-6)
Supplement: Supplementary file 1 — Supplementary material 1 (DOCX 69 kb) [file 10654_2015_106_MOESM1_ESM.docx]

**Supplementary material**

**Body mass index, gestational weight gain and fatty acid concentrations during pregnancy. The Generation R Study**

Aleksandra Jelena Vidakovic MD^1,2,3^, Vincent WV Jaddoe MD, PhD^1,2,3^, Olta Gishti MD, PhD^1,2,3^, Janine F. Felix MD, PhD^1,2,3^, Michelle A. Williams PhD^5^, Albert Hofman MD, PhD^3^, Hans Demmelmair PhD^6^, Berthold Koletzko MD, PhD Dr h.c.^6^, Henning Tiemeier MD, PhD^3,4^ ,

Romy Gaillard PhD*^1,2,3^

1.The Generation R Study Group, Erasmus MC, University Medical Center, Rotterdam, the Netherlands; 2. Department of Pediatrics, Erasmus MC, University Medical Center, Rotterdam, the Netherlands; 3. Department of Epidemiology, Erasmus MC, University Medical Center, Rotterdam, the Netherlands; 4. Department of Child and Adolescent Psychiatry, Erasmus MC, University Medical Center, Rotterdam, the Netherlands; 5. Harvard T.H. Chan School of Public Health, Boston, MA, USA; 6. Div. Metabolic Medicine, Department of Pediatrics, Dr. von Hauner Children’s Hospital, Ludwig-Maximilians-University of Munich Medical Center, München, Germany.

*Correspondence to: Romy Gaillard, PhD. The Generation R Study Group (Na29-15). Erasmus MC, University Medical Center, PO Box 2040, 3000 CA Rotterdam, the Netherlands. (r.gaillard@erasmusmc.nl). Phone: +31 (0) 10 7043405, Fax: +31 (0) 10 7044645

**Table S1** Correlation between all maternal fatty acid concentrations^a^

| Fatty acids | Total SFA | 14:0 | 16:0 | 18:0 | Total MUFAs | 16:1n7 | 18:1n9 | Total n-3 PUFA | 18:3n3 | 20:5n3 | 22:6n3 | Total n-6 PUFA | 18:2n6 | 20:3n6 | 20:4n6 |
| --- | --- | --- | --- | --- | --- | --- | --- | --- | --- | --- | --- | --- | --- | --- | --- |
| Total SFA | 1 |  |  |  |  |  |  |  |  |  |  |  |  |  |  |
| 14:0 | 0.59* | 1 |  |  |  |  |  |  |  |  |  |  |  |  |  |
| 16:0 | 0.98* | 0.59* | 1 |  |  |  |  |  |  |  |  |  |  |  |  |
| 18:0 | 0.87* | 0.41* | 0.78* | 1 |  |  |  |  |  |  |  |  |  |  |  |
| Total MUFA | 0.79* | 0.57* | 0.81* | 0.60* | 1 |  |  |  |  |  |  |  |  |  |  |
| 16:1n7 | 0.60* | 0.63* | 0.65* | 0.33* | 0.73* | 1 |  |  |  |  |  |  |  |  |  |
| 18:1n9 | 0.77* | 0.55* | 0.78* | 0.60* | 0.99* | 0.66* | 1 |  |  |  |  |  |  |  |  |
| Total n-3 PUFA | 0.51* | 0.28* | 0.51* | 0.42* | 0.45* | 0.26* | 0.43* | 1 |  |  |  |  |  |  |  |
| 18:3n3 | 0.44* | 0.44* | 0.44* | 0.35* | 0.52* | 0.35* | 0.52* | 0.34* | 1 |  |  |  |  |  |  |
| 20:5n3 | 0.28* | 0.24* | 0.29* | 0.20* | 0.29* | 0.21* | 0.28* | 0.77* | 0.28* | 1 |  |  |  |  |  |
| 22:6n3 | 0.46* | 0.20* | 0.47* | 0.39* | 0.38* | 0.18* | 0.36* | 0.97* | 0.21* | 0.64* | 1 |  |  |  |  |
| Total n-6 PUFA | 0.85* | 0.34* | 0.80* | 0.86* | 0.50* | 0.28* | 0.50* | 0.22* | 0.29* | 0.00 | 0.21* | 1 |  |  |  |
| 18:2n6 | 0.64* | 0.23* | 0.59* | 0.69* | 0.32* | 0.05* | 0.33* | 0.08* | 0.31* | -0.07* | 0.07* | 0.87* | 1 |  |  |
| 20:3n6 | 0.74* | 0.52* | 0.75* | 0.59* | 0.65* | 0.66* | 0.62* | 0.23* | 0.32* | 0.11* | 0.18* | 0.61* | 0.35* | 1 |  |
| 20:4n6 | 0.59* | 0.14* | 0.57* | 0.59* | 0.34* | 0.23* | 0.31* | 0.32* | -0.01 | 0.11* | 0.35* | 0.63* | 0.22* | 0.35* | 1 |

^a^Values are Pearson correlation coefficients. *P- value <0.06.

**Table S2** Maternal mid-pregnancy fatty acid levels according to body mass index (N=5,636)^a^

|  | Total Group  (5,636) | Underweight  [<20.0 kg/m^2^]  (n=881) | Normal weight  [20.0-24.9 kg/m^2^]  (n=3,162) | Overweight  [25.0-29.9 kg/m^2^]  (n=1,094) | Obesity  [≥30.0 kg/m^2^]  (n=499) | *P* Value* |
| --- | --- | --- | --- | --- | --- | --- |
| *Saturated fatty acids (SF)* |  |  |  |  |  |  |
| Total SFA, mg/L | 696.3 (102.4) | 677.8 (98.4) | 698.5 (102.4) | 702.6 (103.4) | 701.1 (103.9) |  |
| Myristic acid (C14:0), mg/L | 10.6 (3.6) | 10.7 (3.7) | 10.9 (3.6) | 10.2 (3.4) | 9.5 (3.5) | <0.01 |
| Palmitic acid (C16:0), mg/L | 494.9 (76.6) | 481.3 (74.0) | 496.7 (76.6) | 499.1 (77.5) | 499.3 (77.1) | <0.01 |
| Stearic acid (C18:0), mg/L | 184.9 (27.8) | 180.1 (26.0) | 185.1 (27.8) | 187.6 (28.7) | 186.9 (28.2) | <0.01 |
| *Monounsaturated fatty acids (MUFAs)* |  |  |  |  |  |  |
| Total MUFA, mg/L | 206.4 (40.1) | 205.8 (39.8) | 208.7 (39.8) | 203.1 (40.7) | 199.9 (40.2) |  |
| Palmitoleic acid (C16:1n7), mg/L | 12.1 (5.5) | 11.5 (5.5) | 12.2 (5.6) | 12.1 (5.4) | 12.8 (5.6) | <0.01 |
| Oleic acid (C18:1n9), mg/L | 23.7 (4.5) | 23.3 (4.3) | 23.9 (4.4) | 23.5 (4.5) | 23.6 (4.7) | <0.01 |
| *n-3 Polyunsaturated fatty acids (PUFAs)* |  |  |  |  |  |  |
| Total n-3 PUFAs, mg/L | 103.8 (27.4) | 101.7 (28.5) | 106.3 (27.7) | 101.4 (27.7) | 96.9 (22.0) | <0.01 |
| α-Linolenic acid (C18:3n3), mg/L | 5.1 (1.9) | 5.3 (2.0) | 5.2 (1.8) | 4.7 (1.8) | 4.4 (1.6) | <0.01 |
| Eicosapentaenoic acid (C20:5n3), mg/L | 8.5 (5.4) | 8.5 (5.7) | 8.9 (5.5) | 8.0 (5.1) | 7.3 (3.9) | <0.01 |
| Docosahexaenoic acid (C22:6n3), mg/L | 76.7 (20.3) | 74.5 (21.1) | 78.2 (20.5) | 75.7 (20.1) | 72.9 (16.8) | <0.01 |
| *n-6 Polyunsaturated fatty acids (PUFAs)* |  |  |  |  |  |  |
| Total n-6 PUFA, mg/L | 602.0 (88.9) | 584.7 (82.8) | 600.7 (88.9) | 614.6 (90.9) | 613.2 (89.5) | <0.01 |
| Linoleic acid (C18:2n6), mg/L | 360.6 (62.5) | 358.4 (59.3) | 361.2 (62.7) | 364.1 (64.6) | 353.1 (60.9) | <0.01 |
| Dihomo-gamma linolenic acid (C20:3n6), mg/L | 60.7 (16.8) | 55.9 (15.3) | 60.7 (16.5) | 62.7 (17.5) | 65.3 (17.6) | <0.01 |
| Arachidonic acid(C20:4n6), mg/L | 155.7 (32.6) | 145.8 (29.3) | 153.8 (31.1) | 162.7 (33.7) | 170.3 (36.7) | <0.01 |
| n6/n3 PUFAs ratio, mg/L | 6.2 (1.7) | 6.2 (1.7) | 6.0 (1.7) | 6.4 (1.8) | 6.6 (1.5) | <0.01 |

^a^Values represent mean (SD).

*Differences in subject characteristics between groups were evaluated using 1-way ANOVA test.

**Table S3** Comparison of subject characteristics according to gestational weight gain (N=2,697)^a^

|  |  | Weight gain categories | | | |
| --- | --- | --- | --- | --- | --- |
|  | Total Group  (2,697) | Insuficient gestational weight gain  (n=546) | Sufficient gestational weight gain (n=922)  (n=936) | Excessive gestational weight gain  (n=1,229) | *P* Value* |
| *Maternal Characteristics* |  |  |  |  |  |
| Age, y | 29.3 (5.6) | 30.7 (5.2) | 31.5 (4.4) | 30.8 (4.7) | 0.02 |
| Height, cm | 169.0 (7.2) | 167.8 (6.9) | 168.9 (7.3) | 169.7 (7.1) | <0.01 |
| Weight, kg | 68.7 (12.1) | 65.6 (11.9) | 65.6 (10.4) | 72.5 (12.6) | <0.01 |
| Body mass index, kg/m^2^ | 23.2 (3.9) | 23.1 (4.3) | 22.2 (3.2) | 23.9 (4.0) | <0.01 |
| Education, N higher education (%) | 1541 (57.9) | 284 (52.8) | 583 (64.2) | 674 (55.5) | <0.01 |
| Race / Ethnicity, European N (%) | 1939 (72.2) | 352 (64.7) | 680 (74) | 907 (74) | <0.01 |
| Parity, N nulliparous (%) | 1635 (60.7) | 292 (53.5) | 534 (57.9) | 809 (65.9) | <0.01 |
| Total energy intake, kcal | 2079 (543) | 2066 (567) | 2043 (554) | 2110 (523) | 0.56 |
| Carbohydrates, Energy% | 48.5 (6.2) | 48.9 (6.1) | 48.1 (6.3) | 48.6 (6.2) | 0.36 |
| Proteins, Energy% | 15.1 (2.6) | 15.2 (2.6) | 15.2 (2.5) | 14.9 (2.6) | 0.49 |
| Fat, Energy% | 36.0 (5.3) | 35.5 (5.4) | 36.2 (5.3) | 36.1 (5.3) | 0.38 |
| Folic acid supplement use (yes), N (%) | 1919 (83.6) | 360 (77.7) | 656 (84.7) | 903 (85.2) | <0.01 |
| Smoking during pregnancy (yes), N (%) | 614 (23.8) | 80 (15.2) | 170 (19.3) | 364 (30.9) | <0.01 |
| Alcohol consumption during pregnancy (yes), N (%) | 1598 (61.9) | 310 (58.8) | 581 (65.9) | 707 (60.3) | 0.02 |

^a^Values represent mean (SD), median (95% range), or number of subjects (valid%).

*Differences in subject characteristics between groups were evaluated using 1-way ANOVA test.

**Table S4** Associations of maternal gestational weight gain during pregnancy with saturated and monounsaturated fatty acid concentrations unadjusted for prepregnancy body mass index (N = 2,697)^a^

|  | Difference in saturated fatty acid (SFA) concentrations (95% Confidence Interval) | | | | | |
| --- | --- | --- | --- | --- | --- | --- |
|  | Total SFAs (SD) | Myristic acid (SD) | | Palmitic acid (SD) | | Stearic acid (SD) |
| Gestational weight gain^b,c^ |  |  | |  | |  |
| Insufficient gestational weight gain | -0.05 (-0.15, 0.06) | -0.28 (-0.38, -0.18)* | | -0.03 (-0.13, 0.07) | | -0.05 (-0.15, 0.06) |
| Sufficient gestational weight gain | *Reference* | *Reference* | | *Reference* | | *Reference* |
| Excessive gestational weight gain | 0.19 (0.10, 0.27)* | 0.11 (0.03, 0.19)* | | 0.17 (0.09, 0.25)* | | 0.20 (0.11, 0.28)* |
|  | Difference in monounsaturated fatty acids (MUFAs) concentrations (95% Confidence Interval) | | | | | |
|  | Total MUFAs (SD) | | Palmitoleic acid (SD) | | Oleic acid (SD) | |
| Gestational weight gain^b,c^ |  | |  | |  | |
| Insufficient gestational weight gain | -0.04 (-0.14, 0.06) | | -0.10 (-0.20, 0) | | -0.05 (-0.15, 0.05) | |
| Sufficient gestational weight gain | *Reference* | | *Reference* | | *Reference* | |
| Excessive gestational weight gain | 0.16 (0.08, 0.24)* | | 0.21 (0.13, 0.30)* | | 0.15 (0.07, 0.23)* | |

^a^Values are regression coefficients (95% Confidence Interval) that reflect the difference in SD of saturated and monounsaturated fatty acid concentrations for insufficient weight gain and excessive weight gain women as compared to women with sufficient weight gain.

^b^Models are adjusted for maternal age, educational level, ethnicity, parity, smoking and alcohol consumption, folic acid supplement use and total caloric and fat intake during pregnancy. * P-value<0.05.

^c^Models are unadjusted for maternal prepregnancy body mass index.

**Table S5** Associations of maternal gestational weight gain during pregnancy with n-3 and n-6 polyunsaturated fatty acid concentrations unadjusted for prepregnancy body mass index (N = 2,697)^a^

|  | Difference in n-3 Polyunsaturated fatty acids (PUFAs) concentrations (95% Confidence Interval) | | | | | |
| --- | --- | --- | --- | --- | --- | --- |
|  | Total n-3 PUFAs (SD) | α-Linolenic acid (SD) | | Eicosapentaenoic acid (SD) | | Docosahexaenoic acid (SD) |
| Gestational weight gain^b,c^ |  |  | |  | |  |
| Insufficient gestational weight gain | -0.07 (-0.18, 0.03) | -0.16 (-0.26, -0.06)* | | -0.18 (-0.29, -0.08)* | | -0.01 (-0.11, 0.09) |
| Sufficient gestational weight gain | *Reference* | *Reference* | | *Reference* | | *Reference* |
| Excessive gestational weight gain | -0.01 (-0.09, 0.07) | -0.01 (-0.09, 0.08) | | -0.01 (-0.09, 0.09) | | -0.03 (-0.11, 0.06) |
|  | Difference in n-6 Polyunsaturated fatty acids (PUFAs) concentrations (95% Confidence Interval) | | | | | |
|  | Total n-6 PUFAs (SD) | | Linoleic acid (SD) | Dihomo-gamma linolenic acid (SD) | Arachidonic acid (SD) | |
| Gestational weight gain^b,c^ |  | |  |  |  | |
| Insufficient gestational weight gain | 0.03 (-0.07, 0.13) | | 0.01 (-0.10, 0.11) | -0.05 (-0.15, 0.05) | 0.10 (-0.02, 0.20) | |
| Sufficient gestational weight gain | *Reference* | | *Reference* | *Reference* | *Reference* | |
| Excessive gestational weight gain | 0.15 (0.07, 0.23)* | | 0.03 (-0.06, 0.11) | 0.36 (0.28, 0.44)* | 0.13 (0.04, 0.22)* | |

^a^Values are regression coefficients (95% Confidence Interval) that reflect the difference in SD of n-3 and n-6 PUFA concentrations for insufficient weight gain and excessive weight gain women as compared to women with sufficient weight gain.

^b^Models are adjusted for maternal age, educational level, ethnicity, parity, smoking and alcohol consumption, folic acid supplement use and total caloric and fat intake during pregnancy. * P-value<0.05.

^c^Models are unadjusted for maternal prepregnancy body mass index.

**Table S6** Associations of maternal weight during pregnancy with saturated fatty acid concentrations unadjusted for total energy and fat intake (N = 5,636)^a,b^

|  | Difference in saturated fatty acid (SFA) concentrations (95% Confidence Interval) | | | |
| --- | --- | --- | --- | --- |
|  | Total SFAs (SD) | Myristic acid (SD) | Palmitic acid (SD) | Stearic acid (SD) |
| Body mass index^c^ |  |  |  |  |
| Underweight [<20.0 kg/m^2^] | -0.17 (-0.24, -0.10)* | -0.03 (-0.10, 0.05) | -0.17 (-0.24, -0.10)* | -0.16 (-0.23, -0.09)* |
| Normal weight [20.0-24.9 kg/m^2^] | *Reference* | *Reference* | *Reference* | *Reference* |
| Overweight [25.0-29.9 kg/m^2^] | 0.07 (0.01, 0.14)* | -0.13 (-0.20, -0.07)* | 0.07 (0.01, 0.14)* | 0.08 (0.02, 0.15)* |
| Obesity [≥30.0 kg/m^2^] | 0.08 (-0.01, 0.18) | -0.29 (-0.38, -0.20)* | 0.11 (0.01, 0.20)* | 0.06 (-0.04, 0.15) |
| *Body mass index (SD)* | *0.07 (0.04, 0.09)** | *-0.09 (-0.11, -0.06)** | *0.07 (0.05, 0.10)** | *0.06 (0.03, 0.09)** |
|  | Difference in saturated fatty acid (SFA) concentrations (95% Confidence Interval) | | | |
|  | Total SFAs (SD) | Myristic acid (SD) | Palmitic acid (SD) | Stearic acid (SD) |
| Gestational weight gain^c^ |  |  |  |  |
| Insufficient gestational weight gain | -0.06 (-0.16, 0.05) | -0.26 (-0.36, -0.16)* | -0.04 (-0.14, 0.06) | -0.06 (-0.16, 0.05) |
| Sufficient gestational weight gain | *Reference* | *Reference* | *Reference* | *Reference* |
| Excessive gestational weight gain | 0.16 (0.08, 0.25)* | 0.15 (0.07, 0.23)* | 0.15 (0.06, 0.23)* | 0.18 (0.09, 0.26)* |
| *Gestational weight gain (SD)^d^* | *0.09 (0.05, 0.13)** | *0.18 (0.14, 0.22)** | *0.08 (0.04, 0.11)** | *0.10 (0.06, 0.14)** |
| *Gestational weight gain (SD)^e^* | *0.10 (0.06, 0.14)** | *0.17 (0.14, 0.21)** | *0.09 (0.05, 0.12)** | *0.11 (0.07, 0.15)** |

^a^Values are regression coefficients (95% Confidence Interval) that reflect the difference in SD of saturated fatty acid concentrations for underweight, overweight and obese women as compared to normal weight women, and per SD increase in prepregnancy body mass index. Models are adjusted for age, educational level, ethnicity, parity, smoking and alcohol consumption and folic acid supplement use during pregnancy. ^c^Models are unadjusted for total energy and fat intake * P-value<0.05.

^b^Values are regression coefficients (95% Confidence Interval) that reflect the difference in SD of saturated fatty acid concentrations for insufficient weight gain and excessive weight gain women as compared to women with sufficient weight gain, and per SD increase in weight gain. Models are adjusted for age, educational level, ethnicity, parity, smoking and alcohol consumption and folic acid supplement use during pregnancy. Models for weight gain defined according to the IOM criteria are additionally adjusted for prepregnancy body mass index. ^c^Models are unadjusted for total energy and fat intake.  ^d^Gestational weight gain SD unadjusted for body mass index. ^e^Gestational weight gain SD adjusted for body mass index. * P-value<0.05.

.

|  | Difference in monounsaturated fatty acids (MUFAs) concentrations (95% Confidence Interval) | | |
| --- | --- | --- | --- |
|  | Total MUFAs (SD) | Palmitoleic acid (SD) | Oleic acid (SD) |
| Body mass index^c^ |  |  |  |
| Underweight [<20.0 kg/m^2^] | -0.05 (-0.12, 0.02) | -0.12 (-0.19, -0.05)* | -0.02 (-0.09, 0.05) |
| Normal weight [20.0-24.9 kg/m^2^] | *Reference* | *Reference* | *Reference* |
| Overweight [25.0-29.9 kg/m^2^] | -0.02 (-0.09, 0.04) | 0.05 (-0.02, 0.12) | -0.03 (-0.10, 0.03) |
| Obesity [≥30.0 kg/m^2^] | -0.04 (-0.13, 0.05) | 0.21 (0.11, 0.30)* | -0.09 (-0.18, 0.01) |
| *Body mass index (SD)* | *-0.01 (-0.03, 0.02)* | *0.08 (0.05, 0.10)** | *-0.02 (-0.05, 0.01)* |
|  | Difference in monounsaturated fatty acids (MUFAs) concentrations (95% Confidence Interval) | | |
|  | Total MUFAs (SD) | Palmitoleic acid (SD) | Oleic acid (SD) |
| Gestational weight gain^c^ |  |  |  |
| Insufficient gestational weight gain | -0.04 (-0.13, 0.06) | -0.11 (-0.21, -0.01)* | -0.04 (-0.14, 0.06) |
| Sufficient gestational weight gain | *Reference* | *Reference* | *Reference* |
| Excessive gestational weight gain | 0.16 (0.08, 0.24)* | 0.18 (0.10, 0.27)* | 0.16 (0.08, 0.24)* |
| *Gestational weight gain (SD)^d^* | *0.10 (0.06, 0.13)** | *0.13 (0.10, 0.17)** | *0.10 (0.06, 0.14)** |
| *Gestational weight gain (SD)^e^* | *0.10 (0.06, 0.14)** | *0.15 (0.11, 0.18)** | *0.10 (0.06, 0.14)** |

**Table S7** Associations of maternal weight during pregnancy with monounsaturated fatty acid concentrations unadjusted for total energy and fat intake (N = 5,636)^a,b^

^a^Values are regression coefficients (95% Confidence Interval) that reflect the difference in SD of monounsaturated fatty acid concentrations for underweight, overweight and obese women as compared to normal weight women, and per SD increase in prepregnancy body mass index. Models are adjusted for age, educational level, ethnicity, parity, smoking and alcohol consumption and folic acid supplement use during pregnancy. ^c^Models are unadjusted for total energy and fat intake.* P-value<0.05. ^b^Values are regression coefficients (95% Confidence Interval) that reflect the difference in SD of monounsaturated fatty acid concentrations for insufficient weight gain, and excessive weight gain women as compared to women with sufficient weight gain, and per SD increase in weight gain. Models are adjusted for age, educational level, ethnicity, parity, smoking and alcohol consumption and folic acid supplement use during pregnancy. ^c^Models are unadjusted for total energy and fat intake. Models for weight gain defined according to the IOM criteria are additionally adjusted for prepregnancy body mass index.

^d^Gestational weight gain SD unadjusted for body mass index. ^e^Gestational weight gain SD adjusted for body mass index.* P-value<0.05.

**Table S8** Associations of maternal weight during pregnancy with n-3 polyunsaturated fatty acid concentrations unadjusted for total energy and fat intake (N = 5,636)^a,b^

|  | Difference in n-3 Polyunsaturated fatty acids (PUFAs) concentrations (95% Confidence Interval) | | | |
| --- | --- | --- | --- | --- |
|  | Total n-3 PUFAs (SD) | α-Linolenic acid (SD) | Eicosapentaenoic acid (SD) | Docosahexaenoic acid (SD) |
| Body mass index^c^ |  |  |  |  |
| Underweight [<20.0 kg/m^2^] | -0.11 (-0.18, -0.05)* | 0.06 (-0.01, 0.13) | -0.03 (-0.10, 0.04) | -0.13 (-0.20, -0.06)* |
| Normal weight [20.0-24.9 kg/m^2^] | *Reference* | *Reference* | *Reference* | *Reference* |
| Overweight [25.0-29.9 kg/m^2^] | -0.04 (-0.11, 0.02) | -0.18 (-0.24, -0.11)* | -0.05 (-0.11, 0.02) | -0.01 (-0.07, 0.06) |
| Obesity [≥30.0 kg/m^2^] | -0.10 (-0.19, -0.02)* | -0.34 (-0.43, -0.25)* | -0.09 (-0.18, 0.01) | -0.04 (-0.13, 0.05) |
| *Body mass index (SD)* | *-0.02 (-0.04, 0.01)* | *-0.13 (-0.15, -0.10)** | *-0.02 (-0.05, 0.01)* | *0.01 (-0.02, 0.03)* |
|  | Difference in n-3 Polyunsaturated fatty acids (PUFAs) concentrations (95% Confidence Interval) | | | |
|  | Total n-3 PUFAs (SD) | α-Linolenic acid (SD) | Eicosapentaenoic acid (SD) | Docosahexaenoic acid (SD) |
| Weight gain^c^ |  |  |  |  |
| Insufficient gestational weight gain | -0.07 (-0.17, 0.04) | -0.14 (-0.24, -0.04)* | -0.18 (-0.28, -0.07)* | -0.01 (-0.11, 0.10) |
| Sufficient gestational weight gain | *Reference* | *Reference* | *Reference* | *Reference* |
| Excessive gestational weight gain | 0.01 (-0.08, 0.09) | 0.05 (-0.03, 0.14) | 0.02 (-0.07, 0.11) | -0.02 (-0.11, 0.06) |
| *Gestational weight gain (SD)^d^* | *0.03 (-0.01, 0.06)* | *0.09 (0.05, 0.13)** | *0.08 (0.04, 0.12)** | *-0.02 (-0.06, 0.02)* |
| *Gestational weight gain (SD)^e^* | *0.02 (-0.02, 0.06)* | *0.07 (0.04, 0.11)** | *0.08 (0.04, 0.12)** | *-0.02 (-0.06, 0.02)* |

^a^Values are regression coefficients (95% Confidence Interval) that reflect the difference in SD of n-3 PUFA concentrations for underweight, overweight and obese women as compared to normal weight women, and per SD increase in prepregnancy body mass index. Models are adjusted for, age, educational level, ethnicity, parity, smoking and alcohol consumption and folic acid supplement use during pregnancy. ^c^Models are unadjusted for total energy and fat intake.* P-value<0.05.

^b^Values are regression coefficients (95% Confidence Interval) that reflect the difference in SD of n-3 PUFA concentrations for insufficient weight gain, and excessive weight gain women as compared to women with sufficient weight gain, and per SD increase in weight gain. Models are adjusted for age, educational level, ethnicity, parity, smoking and alcohol consumption and folic acid supplement use during pregnancy. ^c^Models are unadjusted for total energy and fat intake. Models for weight gain defined according to the IOM criteria are additionally adjusted for prepregnancy body mass index. ^d^Gestational weight gain SD unadjusted for body mass index. ^e^Gestational weight gain SD adjusted for body mass index.* P-value<0.05.

**Table S9** Associations of maternal weight during pregnancy with n-6 polyunsaturated fatty acid concentrations unadjusted for total energy and fat intake (N = 5,636)^a,b^

|  | Difference in n-6 Polyunsaturated fatty acids (PUFAs) concentrations (95% Confidence Interval) | | | |
| --- | --- | --- | --- | --- |
|  | Total n-6 PUFAs (SD) | Linoleic acid (SD) | Dihomo-gamma linolenic acid (SD) | Arachidonic acid (SD) |
| Body mass index^c^ |  |  |  |  |
| Underweight [<20.0 kg/m^2^] | -0.16 (-0.24, -0.09)* | -0.03 (-0.10, 0.04) | -0.27 (-0.34, -0.20)* | -0.23 (-0.30, -0.16)* |
| Normal weight [20.0-24.9 kg/m^2^] | *Reference* | *Reference* | *Reference* | *Reference* |
| Overweight [25.0-29.9 kg/m^2^] | 0.09 (0.02, 0.16)* | -0.02 (-0.09, 0.05) | 0.16 (0.09, 0.23)* | 0.23 (0.16, 0.30)* |
| Obesity [≥30.0 kg/m^2^] | 0.05 (-0.05, 0.14) | -0.23 (-0.32, -0.13)* | 0.34 (0.24, 0.43)* | 0.44 (0.34, 0.53)* |
| *Body mass index (SD)* | *0.06 (0.03, 0.09)** | *-0.05 (-0.08, -0.02)** | *0.16 (0.13, 0.18)** | *0.19 (0.16, 0.22)** |
|  | Difference in n-6 Polyunsaturated fatty acids (PUFAs) concentrations (95% Confidence Interval) | | | |
|  | Total n-6 PUFAs (SD) | Linoleic acid (SD) | Dihomo-gamma linolenic acid (SD) | Arachidonic acid (SD) |
| Weight gain^c^ |  |  |  |  |
| Insufficient gestational weight gain | 0.02 (-0.09, 0.12) | 0.01 (-0.09, 0.11) | -0.08 (-0.18, 0.02) | 0.07 (-0.03, 0.17) |
| Sufficient gestational weight gain | *Reference* | *Reference* | *Reference* | *Reference* |
| Excessive gestational weight gain | 0.13 (0.04, 0.21)* | 0.05 (-0.04, 0.14) | 0.29 (0.21, 0.38)* | 0.05 (-0.03, 0.13) |
| *Gestational weight gain (SD)^d^* | *0.04 (0.01, 0.08)** | *0.02 (-0.02, 0.06)* | *0.15 (0.11, 0.19)** | *-0.03 (-0.06, 0.01)* |
| *Gestational weight gain (SD)^e^* | *0.05 (0.02, 0.09)** | *0.01 (-0.02, 0.05)* | *0.18 (0.14, 0.22)** | *-0.00 (-0.04, 0.04)* |

^a^Values are regression coefficients (95% Confidence Interval) that reflect the difference in SD of n-6 PUFA concentrations for underweight, overweight and obese women as compared to normal weight women, and per SD increase in prepregnancy body mass index. Models are adjusted for, age, educational level, ethnicity, parity, smoking and alcohol consumption and folic acid supplement use during pregnancy. ^c^Models are unadjusted for total energy and fat intake.* P-value<0.05.

^b^Values are regression coefficients (95% Confidence Interval) that reflect the difference in SD of n-6 PUFA concentrations for insufficient weight gain, and excessive weight gain women as compared to women with sufficient weight gain, and per SD increase in weight gain. Models are adjusted for maternal body mass index, age, educational level, ethnicity, parity, smoking and alcohol consumption and folic acid supplement use during pregnancy. ^c^Models are unadjusted for total energy and fat intake. Models for weight defined according to the IOM criteria are additionally adjusted for prepregnancy body mass index. ^d^Gestational weight gain SD unadjusted for body mass index. ^e^Gestational weight gain SD adjusted for body mass index.* P-value<0.05.

**Table S10** Associations of maternal weight during pregnancy with saturated fatty acid concentrations unadjusted for maternal characteristics (N = 5,636)^a,b^

|  | Difference in saturated fatty acid (SFA) concentrations (95% Confidence Interval) | | | |
| --- | --- | --- | --- | --- |
|  | Total SFAs (SD) | Myristic acid (SD) | Palmitic acid (SD) | Stearic acid (SD) |
| Body mass index^c^ |  |  |  |  |
| Underweight [<20.0 kg/m^2^] | -0.20 (-0.28, -0.13)* | -0.05 (-0.12, 0.02) | -0.20 (-0.28, -0.13)* | -0.18 (-0.25, -0.10)* |
| Normal weight [20.0-24.9 kg/m^2^] | *Reference* | *Reference* | *Reference* | *Reference* |
| Overweight [25.0-29.9 kg/m^2^] | 0.04 (-0.03, 0.11)* | -0.19 (-0.26, -0.12)* | 0.03 (-0.04, 0.10) | 0.09 (0.02, 0.16)* |
| Obesity [≥30.0 kg/m^2^] | 0.03 (-0.06, 0.12)* | -0.39 (-0.48, -0.29)* | 0.03 (-0.06, 0.13) | 0.07 (-0.03, 0.16) |
| *Body mass index (SD)* | *0.06 (0.03, 0.08)** | *-0.11 (-0.14, -0.09)** | *0.06 (0.03, 0.08)** | *0.07 (0.04. 0.09)** |
|  | Difference in saturated fatty acid (SFA) concentrations (95% Confidence Interval) | | | |
|  | Total SFAs (SD) | Myristic acid (SD) | Palmitic acid (SD) | Stearic acid (SD) |
| Gestational weight gain^c^ |  |  |  |  |
| Insufficient gestational weight gain | -0.08 (-0.18, 0.02) | -0.30 (-0.40, -0.20)* | -0.07 (-0.17, 0.03) | -0.06 (-0.16, 0.04) |
| Sufficient gestational weight gain | *Reference* | *Reference* | *Reference* | *Reference* |
| Excessive gestational weight gain | 0.17 (0.09, 0.25)* | 0.16 (0.08, 0.24)* | 0.15 (0.07, 0.23)* | 0.19 (0.10, 0.27)* |
| *Gestational weight gain (SD)^d^* | *0.09 (0.05, 0.13)** | *0.18 (0.14, 0.22)** | *0.08 (0.04, 0.11)** | *0.10 (0.06, 0.14)** |
| *Gestational weight gain (SD)^e^* | *0.10 (0.06, 0.14)** | *0.17 (0.13, 0.21)** | *0.09 (0.05, 0.12)** | *0.11 (0.07, 0.15)** |

^a^Values are regression coefficients (95% Confidence Interval) that reflect the difference in SD of saturated fatty acid concentrations for underweight, overweight and obese women as compared to normal weight women, and per SD increase in prepregnancy body mass index.

Models are unadjusted for potential confounders. * P-value<0.05.

^b^Values are regression coefficients (95% Confidence Interval) that reflect the difference in SD of saturated fatty acid concentrations for insufficient weight gain and excessive weight gain women as compared to women with sufficient weight gain, and per SD increase in weight gain.

^c^Models are unadjusted for potential confounders.

Models for weight gain defined according to the IOM criteria are additionally adjusted for prepregnancy body mass index. ^d^Gestational weight gain SD unadjusted for body mass index. ^e^Gestational weight gain SD adjusted for body mass index. * P-value<0.05.

**Table S11** Associations of maternal weight during pregnancy with monounsaturated fatty acid concentrations unadjusted for maternal characteristics (N = 5,636)^a,b^

|  | Difference in monounsaturated fatty acids (MUFAs) concentrations (95% Confidence Interval) | | |
| --- | --- | --- | --- |
|  | Total MUFAs (SD) | Palmitoleic acid (SD) | Oleic acid (SD) |
| Body mass index^c^ |  |  |  |
| Underweight [<20.0 kg/m^2^] | -0.06 (-0.14, 0.01) | -0.12 (-0.19, -0.05)* | -0.05 (-0.12, 0.03) |
| Normal weight [20.0-24.9 kg/m^2^] | *Reference* | *Reference* | *Reference* |
| Overweight [25.0-29.9 kg/m^2^] | -0.14 (-0.20, -0.07)* | -0.02 (-0.09, 0.05) | -0.15 (-0.22, -0.08)* |
| Obesity [≥30.0 kg/m^2^] | -0.22 (-0.31, -0.12)* | 0.11 (0.01, 0.20)* | -0.26 (-0.36, -0.17)* |
| *Body mass index (SD)* | *-0.06 (-0.08, -0.03)** | *0.05 (0.02, 0.07)** | *-0.07 (-0.10, -0.05)** |
|  | Difference in monounsaturated fatty acids (MUFAs) concentrations (95% Confidence Interval) | | |
|  | Total MUFAs (SD) | Palmitoleic acid (SD) | Oleic acid (SD) |
| Gestational weight gain^c^ |  |  |  |
| Insufficient gestational weight gain | -0.10 (-0.20, 0) | -0.15 (-0.25, -0.05)* | -0.10 (-0.20, 0) |
| Sufficient gestational weight gain | *Reference* | *Reference* | *Reference* |
| Excessive gestational weight gain | 0.17 (0.09, 0.26)* | 0.22 (0.14, 0.31)* | 0.17 (0.09, 0.25)* |
| *Gestational weight gain (SD)^d^* | *0.10 (0.06, 0.13)** | *0.13 (0.10, 0.17)** | *0.10 (0.06, 0.14)** |
| *Gestational weight gain (SD)^e^* | *0.10 (0.06, 0.14)** | *0.15 (0.11, 0.19)** | *0.10 (0.06, 0.14)** |

^a^Values are regression coefficients (95% Confidence Interval) that reflect the difference in SD of monounsaturated fatty acid concentrations for underweight, overweight and obese women as compared to normal weight women, and per SD increase in prepregnancy body mass index.

^c^Models are unadjusted for potential confounders. * P-value<0.05.

^b^Values are regression coefficients (95% Confidence Interval) that reflect the difference in SD of monounsaturated fatty acid concentrations for insufficient weight gain and excessive weight gain women as compared to women with sufficient weight gain, and per SD increase in weight gain.

^c^Models are unadjusted for potential confounders. Models for weight gain defined according to the IOM criteria are additionally adjusted for prepregnancy body mass index. ^d^Gestational weight gain SD unadjusted for body mass index. ^e^Gestational weight gain SD adjusted for body mass index. * P-value<0.05.

**Table S12** Associations of maternal weight during pregnancy with n-3 polyunsaturated fatty acid concentrations unadjusted for maternal characteristics (N = 5,636)^a,b^

|  | Difference in n-3 Polyunsaturated fatty acids (PUFAs) concentrations (95% Confidence Interval) | | | |
| --- | --- | --- | --- | --- |
|  | Total n-3 PUFAs (SD) | α-Linolenic acid (SD) | Eicosapentaenoic acid (SD) | Docosahexaenoic acid (SD) |
| Body mass index^c^ |  |  |  |  |
| Underweight [<20.0 kg/m^2^] | -0.16 (-0.24, -0.09)* | 0.04 (-0.03, 0.12) | -0.07 (-0.15, 0.01) | -0.18 (-0.26, -0.11)* |
| Normal weight [20.0-24.9 kg/m^2^] | *Reference* | *Reference* | *Reference* | *Reference* |
| Overweight [25.0-29.9 kg/m^2^] | -0.16 (-0.23, -0.09)* | -0.25 (-0.32, -0.18)* | -0.17 (-0.23, -0.10)* | -0.13 (-0.19, -0.06)* |
| Obesity [≥30.0 kg/m^2^] | -0.32 (-0.41, -0.22)* | -0.44 (-0.54, -0.35)* | -0.30 (-0.40, -0.21)* | -0.26 (-0.36, -0.17)* |
| *Body mass index (SD)* | *-0.07 (-0.10, -0.05)** | *-0.16 (-0.18, -0.13)** | *-0.08 (-0.11, -0.06)** | *-0.05 (-0.08, -0.02)** |
|  | Difference in n-3 Polyunsaturated fatty acids (PUFAs) concentrations (95% Confidence Interval) | | | |
|  | Total n-3 PUFAs (SD) | α-Linolenic acid (SD) | Eicosapentaenoic acid (SD) | Docosahexaenoic acid (SD) |
| Weight gain^c^ |  |  |  |  |
| Insufficient gestational weight gain | -0.15 (-0.25, -0.04)* | -0.18 (-0.28, -0.07)* | -0.25 (-0.36, -0.14)* | -0.08 (-0.19, 0.03) |
| Sufficient gestational weight gain | *Reference* | *Reference* | *Reference* | *Reference* |
| Excessive gestational weight gain | -0.01 (-0.10, 0.08) | 0.07 (-0.01, 0.15) | 0.01 (-0.08, 0.10) | -0.04 (-0.13, 0.05) |
| *Gestational weight gain (SD)^d^* | *0.03 (-0.01, 0.06)* | *0.09 (0.05, 0.13)** | *0.08 (0.04, 0.12)** | *-0.02 (-0.06, 0.02)* |
| *Gestational weight gain (SD)^e^* | *0.02 (-0.02, 0.06)* | *0.07 (0.04, 0.11)** | *0.08 (0.04, 0.12)** | *-0.02 (-0.06, 0.02)* |

^a^Values are regression coefficients (95% Confidence Interval) that reflect the difference in SD of n-3 PUFA concentrations for underweight, overweight and obese women as compared to normal weight women, and per SD increase in prepregnancy body mass index.

^c^Models are unadjusted for potential confounders. *P-value<0.05.

^b^Values are regression coefficients (95% Confidence Interval) that reflect the difference in SD of n-3 PUFA concentrations for insufficient weight gain, and excessive weight gain women as compared to women with sufficient weight gain, and per SD increase in weight gain.

^c^Models are unadjusted for potential confounders. Models for weight gain defined according to the IOM criteria are additionally adjusted for prepregnancy body mass index. ^c^Gestational weight gain SD unadjusted for body mass index. ^d^Gestational weight gain SD adjusted for body mass index.* P-value<0.05.

**Table S13** Associations of maternal weight during pregnancy with n-6 polyunsaturated fatty acid concentrations unadjusted for maternal characteristics (N = 5,636)^a,b^

|  | Difference in n-6 Polyunsaturated fatty acids (PUFAs) concentrations (95% Confidence Interval) | | | |
| --- | --- | --- | --- | --- |
|  | Total n-6 PUFAs (SD) | Linoleic acid (SD) | Dihomo-gamma linolenic acid (SD) | Arachidonic acid (SD) |
| Body mass index^c^ |  |  |  |  |
| Underweight [<20.0 kg/m^2^] | -0.18 (-0.26, -0.11)* | -0.04 (-0.12, 0.03) | -0.28 (-0.36, -0.21)* | -0.25 (-0.32, -0.17)* |
| Normal weight [20.0-24.9 kg/m^2^] | *Reference* | *Reference* | *Reference* | *Reference* |
| Overweight [25.0-29.9 kg/m^2^] | 0.16 (0.10, 0.23)* | 0.05 (-0.02, 0.11) | 0.12 (0.05, 0.19)* | 0.28 (0.21, 0.34)* |
| Obesity [≥30.0 kg/m^2^] | 0.15 (0.06, 0.25)* | -0.13 (-0.22, -0.04)* | 0.28 (0.18, 0.37)* | 0.51 (0.42, 0.60)* |
| *Body mass index (SD)* | *0.10 (0.08, 0.13)** | *-0.01 (-0.04, 0.01)* | *0.14 (0.12, 0.17)** | *0.22 (0.19, 0.24)** |
|  | Difference in n-6 Polyunsaturated fatty acids (PUFAs) concentrations (95% Confidence Interval) | | | |
|  | Total n-6 PUFAs (SD) | Linoleic acid (SD) | Dihomo-gamma linolenic acid (SD) | Arachidonic acid (SD) |
| Weight gain^c^ |  |  |  |  |
| Insufficient gestational weight gain | 0.05 (-0.06, 0.15) | 0.05 (-0.06, 0.15) | -0.10 (-0.19, 0.01) | 0.08 (-0.02, 0.18) |
| Sufficient gestational weight gain | *Reference* | *Reference* | *Reference* | *Reference* |
| Excessive gestational weight gain | 0.13 (0.04, 0.21)* | 0.05 (-0.04, 0.13) | 0.31 (0.23, 0.39)* | 0.06 (-0.03, 0.14) |
| *Gestational weight gain (SD)^d^* | *0.04 (0.01, 0.08)** | *0.02 (-0.02, 0.06)* | *0.15 (0.11, 0.19)** | *-0.03 (-0.06, 0.01)* |
| *Gestational weight gain (SD)^e^* | *0.05 (0.02, 0.09)** | *0.01 (-0.02, 0.05)* | *0.18 (0.14, 0.22)** | *-0.00 (-0.04, 0.04)* |

^a^Values are regression coefficients (95% Confidence Interval) that reflect the difference in SD of n-6 PUFA concentrations for underweight, overweight and obese women as compared to normal weight women, and per SD increase in prepregnancy body mass index.

^c^Models are unadjusted for potential confounders. *P-value<0.05.

^b^Values are regression coefficients (95% Confidence Interval) that reflect the difference in SD of n-6 PUFA concentrations for insufficient weight gain, and excessive weight gain women as compared to women with sufficient weight gain, and per SD increase in weight gain.

^c^Models are unadjusted for potential confounders. Models for weight defined according to the IOM criteria are additionally adjusted for prepregnancy body mass index. ^c^Gestational weight gain SD unadjusted for body mass index. ^d^Gestational weight gain SD adjusted for body mass index.* P-value<0.05.
